# Endophenotypes for Age-Related Macular Degeneration: Extending Our Reach into the Preclinical Stages of Disease

**DOI:** 10.3390/jcm3041335

**Published:** 2014-11-28

**Authors:** Michael B. Gorin, Daniel E. Weeks, Robert V. Baron, Yvette P. Conley, Maria C. Ortube, Steven Nusinowitz

**Affiliations:** 1 Department of Ophthalmology, David Geffen School of Medicine—UCLA, Stein Eye Institute, Los Angeles, CA 90095, USA; E-Mails: ortube@jsei.ucla.edu (M.C.O.); nusinowitz@jsei.ucla.edu (S.N.); 2 Departments of Human Genetics and Biostatistics, Graduate School of Public Health, University of Pittsburgh, Pittsburgh, PA 15261, USA; E-Mail: weeks@pitt.edu; 3 Department of Human Genetics, Graduate School of Public Health, University of Pittsburgh, Pittsburgh, PA 15261, USA; E-Mail: rvb5@pitt.edu; 4 Department of Health Promotion, School of Nursing, University of Pittsburgh, Pittsburgh, PA 15213, USA; E-Mail: yconley@pitt.edu

**Keywords:** age-related macular degeneration, endophenotype, genetic risk, preclinical diagnostics, retinal function, predictive modeling

## Abstract

The key to reducing the individual and societal burden of age-related macular degeneration (AMD)-related vision loss, is to be able to initiate therapies that slow or halt the progression at a point that will yield the maximum benefit while minimizing personal risk and cost. There is a critical need to find clinical markers that, when combined with the specificity of genetic testing, will identify individuals at the earliest stages of AMD who would benefit from preventive therapies. These clinical markers are endophenotypes for AMD, present in those who are likely to develop AMD, as well as in those who have clinical evidence of AMD. Clinical characteristics associated with AMD may also be possible endophenotypes if they can be detected before or at the earliest stages of the condition, but we and others have shown that this may not always be valid. Several studies have suggested that dynamic changes in rhodopsin regeneration (dark adaptation kinetics and/or critical flicker fusion frequencies) may be more subtle indicators of AMD-associated early retinal dysfunction. One can test for the relevance of these measures using genetic risk profiles based on known genetic risk variants. These functional measures may improve the sensitivity and specificity of predictive models for AMD and may also serve to delineate clinical subtypes of AMD that may differ with respect to prognosis and treatment.

## 1. Introduction

In the past ten years, there has been an explosion in our understanding of the genetics of age-related macular degeneration resulting from the initial association of age-related macular degeneration (AMD) with variants of apoE [1], followed by the family-based linkage studies that identified multiple loci for advanced AMD. Two of the major loci that were reported in several family-based linkage studies, 1q31 and 10q26 were the subject of candidate gene testing of variants in both family-based and case-control cohorts and led to the discovery of the roles of complement factor H (CFH) [2,3,4,5] and the *ARMS2/HTRA1* genes [6,7] in AMD risk. At approximately the same time, two genome-wide association studies (GWA) studies [8,9] also identified associations of variants in these two genes with AMD. Multiple candidate gene association studies were undertaken (for review see: [10]) particularly of the genes regulating the alternative complement pathway, confirming risk alleles in C2/BF, C3, and others [11]. Later, large-scale GWA studies with case-control cohorts confirmed these associations as well as with other genes in the complement pathway, lipid metabolism pathways and extracellular matrix biosynthesis and regulation. In addition, several genetic loci that were novel with respect to these pathways have also been found [10]. At present, 20 autosomal genetic loci have common variants with statistically significant associations with AMD. Mitochondrial genetic variants [12,13,14,15,16] as well as rare variants in several genes, notably *CFH*, *CFI*, *C9* and *C3* [17,18,19,20,21], have been implicated in AMD pathogenesis.

Yet, with all these discoveries, we still cannot account for the complete heritability of AMD. There are individuals across the spectrum of AMD risk who will develop the condition despite relatively low genetic risk and others who will not progress to advanced disease, even with several high-risk variants. Further, our risk prediction models are inadequate for use in the preclinical AMD population. Some of this may be due to epigenetic phenomena that have not yet been considered, gene-gene interactions or the effects of other exogenous contributors such as smoking and diet as well as the human microbiome. Further genetic studies may refine our risk models, but it will remain a challenge to know how to interpret the impact of rare variants on genes associated with AMD, particularly when those variants do not affect protein structure.

If one of our primary goals in AMD research is to delineate the pathobiology of the condition, then we are well on our way to achieving that goal, though it will take more than population-based genetic association studies to understanding the underlying biological interactions and pathways. It is important to remember that complex genetic conditions are not simply heterogenous collections of disorders, nor do they follow the classic laws of causality that are attributed to Mendelian genetic disorders. They represent relatively subtle derangements of normal biological processes that over time lead to a dysregulated state associated with a disease process [10]. Some rare variants related to AMD may have sufficient penetrance to mimic a Mendelian genetic disorder [18,19,20], but most of the common variants appear to have a probabilistic impact on disease risk rather than being necessary and sufficient.

If we are hoping to use the molecular genetics of AMD as part of personalized medicine with the intention of the early recognition of at-risk individuals to prevent or reduce the impact of disease, then we need to combine genetics with another set of tools to achieve the sensitivity and specificity to appropriately target the population for treatment. These tools are either to detect preclinical functional and/or structural derangements in the retina/RPE/choroid complex or to measure dynamic levels of molecules in the blood or eye (biomarkers) that are associated with AMD and which reflect both genetic and exogenous influences on pathways that contribute to AMD pathogenesis. There is obvious overlap of these two approaches since instruments that can measure levels of specific molecules in the eye and localize them are both defining structural changes (at a molecular level) in the retina and at the same time are employing the classical concept of measuring a biomarker with respect to measuring quantitative levels of a specific molecule. For structural and functional changes in the retina and biomarkers, we are striving for sensitivity without a specific requisite of specificity since it is unreasonable to expect any given pathway to only be reflected in AMD pathogenesis. For example serum C-reactive protein levels have been shown to be useful in risk models for heart disease but they are also altered in people with an array of autoimmune or infectious disorders [22,23]. Light or dark adaptation kinetics of the retina might be useful indicators of early AMD-related retinal dysfunction but they certainly are abnormal in many disease states that are unrelated to AMD.

This paper examines and tests the concepts of how to use these tools in a complementary fashion with molecular genetics of AMD in order to achieve a clinically relevant predictive risk model. We must accept at the outset that such a model will be imperfect. It will be heavily influenced by the age of the individual and by other co-morbidities that share pathways that interact with AMD pathogenesis. The need for such a risk model is really predicated on the presumption that we will someday have a preventive therapy for AMD and that therapy will have costs and risks that make it appropriate to limit its use to those who are more likely to develop AMD. As long as our preventive options are avoidance of smoking, dietary modifications and supplements, then genetic testing has limited clinical relevance. However, given the efforts of the modern pharmaceutical industry to find an effective therapy for the large number of individuals at risk for AMD and the value of preserving sight in the elderly population (who are an increasing percentage of our population), then it is likely that therapies will emerge, first for those with clinically evident AMD and then extended to those with milder clinical or preclinical findings.

In addition, the timing of when to institute a preventive therapy will be crucial since such treatment will inevitably alter normal physiologic pathways and thus not necessarily be desirable for an entire lifetime. At the same time, we want to start therapy sufficiently early in the disease to have optimal impact in preventing vision compromise. Thus it makes sense to have a process that can inform us as to when to initiate therapy, as well as to monitor a biological response to that therapy (e.g., lowering of the risk for AMD incidence and/or progression) before disease is more clinically evident.

Most clinicians have a strong intuitive sense of what constitutes a disease phenotype. Disease phenotypes are those observable features that are attributable to a specific condition [24]. They may represent the effects of a genetic mutation, genetic modifiers and/or exogenous factors. A single disease can have multiple phenotypic features and a specific phenotypic feature may be present in a number of conditions. For example, with AMD, there are a variety of clinically distinct forms of drusen, each of which can be considered a phenotypic feature [25,26,27,28,29,30]. At the same time a choroidal neovascular membrane (which is part of the definition of exudative AMD) is a phenotypic feature seen in a variety of ocular conditions. When multiple phenotypic features are present, it is possible that one or more may be obscured by another and thus phenotypic features of a disease may emerge or become obscured during the course of a disease. From a genetics standpoint, the appearance of the disease for that individual can change, but the phenotypic features that are observable at any time during the timeframe of the disease are what constitute the phenotype of that person’s condition. Phenotypic features not only define the clinical spectrum of the disease entity but they can play a critical role in predicting the rate of progression and likelihood of advanced disease. Examples of this for AMD would include large soft drusen and serous pigment epithelial detachments that are associated with an increased risk of developing advanced, exudative AMD.

What is an endophenotype? The concept of endophenotype has several origins, but the term really gained acceptance in the psychiatric genetic literature [24,31], when clinicians, faced with the complexity of mental illness, were striving to find measurable traits that were associated with the disease, found in higher prevalence within families of individuals with that disease and which could serve as a surrogate when the clinical definition of the disease could not be met. Thus unlike a phenotype, an endophenotype can be measured or assessed even when the clinical state of disease is not present. To make the association with the disease in the first place, the endophenotype should be measurable in affected individuals (thus it can act as a disease phenotypic feature). It may not be present in all manifestations of the disease and throughout the lifetime of the condition, and it may have predictive value or not as to the future course or progression of the condition. However to be useful as an endophenotype (and not just as a disease phenotype), it must be measureable in clinically unaffected individuals and still be associated with disease. Note that a phenotypic feature that is associated with the disease doesn’t necessarily qualify as an endophenotype, unless it is observed in a higher frequency in family members of an affected individual in comparison with the frequency in the general population [31].

In a sense, the most obvious endophenotype for AMD is the combination of the genetic risk variants themselves. As we have already discussed, a genetic risk model for AMD by itself is imperfect because there are a substantial number of individuals, particularly those with moderate risk profiles who will not eventually develop AMD. However as a group, one can look at cohorts defined by high and low genetic risks for AMD and have a reasonable expectation that a substantially higher percentage of individuals in the high-risk group will develop AMD as compared to the low-risk group. The genetic risk profile itself can serve as an endophenotype for AMD to then test whether or not other phenotypic features of AMD can also meet the definition of an endophenotype. If we have a functional test of the retina, such as dark-adapted thresholds or dark adaptation kinetics that we observe to be abnormal in a significant number of AMD cases, it is reasonable to consider these abnormalities to be phenotypic features of AMD [32,33,34,35,36,37,38,39]. It doesn’t have to be universally observed and they may be useful for distinguishing subtypes of AMD. However to test whether or not these functional measures can serve as endophenotypes, we have to first show that they are abnormal in a higher percentage of the younger at-risk family members of affected individuals than would be observed in the general population. One can test this hypothesis directly by conducting a family-based study of adult children with one or more parents with AMD and using a population-based control group (such as the spouses of those adult children). Alternatively one can construct a case-control study design using the genetic risk profile of individuals as determined from the multiple common variants associated with AMD. This latter approach is much simpler since one doesn’t have to recruit within families and virtually every individual who is tested for the functional state can contribute to the analysis based on their AMD genetic risk profile. Another challenge in using the family-based approach is ensuring that one can effectively recruit a suitable control population since there may be a hidden genetic bias if one exclusively uses relatives (many of whom are presumably presymptomatic), but we have found that recruiting normal adults (ages 50–65) with no family history of AMD for functional testing is far more difficult than enlisting individuals who have a heightened awareness of AMD because of one or more affected parents. While both strategies can be used to test the suitability of a phenotypic feature to be an endophenotype for AMD [40], they are not identical. A recent paper by Aiyar *et al.* [41] showed a poor correlation of a personal family history with genomic testing for a complex disorder such as AMD. Their conclusion is that the discordance suggests that these two approaches for determining familial risk incorporate overlapping information and are partly complementary. They recommend the incorporation of both genomic information and a family history to improve risk assessment.

Clinicians often consider potential AMD endophenotypes in three categories—structural, functional and molecular, but we should recognize that these distinctions are based more on acquisition technologies rather than on any conceptual distinctions. Most of the literature refers to biomarkers as in the measurement of small molecules in the blood (lipids, proteins, solutes), which reflect a systemic state. However biomarkers can also be localized, such as the measurements of retinal autofluorescence for A2E [42], intrinsic fluorescence [43], or macular carotenoids [44,45] with fundus imaging. Redox-based imaging based on differential fluorescence of NAD/NADP and NADH/NADHP in the cornea [46] or oxidized hemoglobin in the brain [47] are additional examples in which functional imaging and biomarker quantitation can be integrated. New noninvasive technologies may make it possible to determine levels of amyloid elements and specific metabolites in the *in vivo* retina/RPE. Functional testing of the retina reflects complex pathways and processing, but under certain conditions, as in a Vitamin A deficiency, can be a surrogate for retinoid availability and processing in the retina and be responsive to vitamin A intake or medications that block vitamin A transport [48].

It is well known that deficits in rod-mediated retinal function occur as a natural consequence of normal aging [36,49,50,51,52] a process that may be accelerated in age-related macular degeneration (AMD) [34,38,39,49,53,54,55,56]. There are numerous psychophysical studies that have demonstrated decreases in rod-mediated sensitivity and prolonged dark-adaptation kinetics even in early AMD [33,34,36,38,39,54,55,56,57,58,59]. Thus, many have proposed scotopic thresholds as a potential endophenotype. Others have suggested that evaluating kinetic processes rather than threshold levels may be more sensitive indicators of early retinal dysfunction [38]. Thus, regional dark adaptometry, which measures the kinetics of rod and cone photoreceptor adaptation are functional tests that may be potential AMD endophenotypes.

As noted above serum biomarkers generally reflect a systemic state that may be indicative of an increased or decreased risk for developing disease. While it easier to gain access to the many metabolites and elements within the blood for large-scale assays, they pose a far greater challenge to relate these biomarkers to AMD than biomarker approaches that are focused on the tissues directly altered by AMD. Given the genes and variants implicated by AMD genetic association studies, one would consider serum biomarkers that reflect the dynamic states of lipid metabolism (e.g., apolipoprotein E), retinoid uptake and transport, carotenoid transport, inflammatory markers including C-reactive protein, circulating complement factors and regulatory proteins, and cytokines. However using these serum biomarkers is problematic in that they reflect complex interactions that include host genetic variants, diet, exogenous exposures, exercise, and the bacterial microbiome and other disease states. Depending on the prevalence of these mitigating factors, a biomarker may be informative for AMD risk in one cohort and not another. To effectively employ serum biomarkers in the general population, one needs a vast amount of data to establish how these molecules vary with age, gender and numerous co-morbidities and exposures so that one can determine if these modifiers contribute to the value of a biomarker for assessing AMD risk or if they confound their use. For example, there is evidence that the risks for heart disease and stroke are lowered by reducing serum cholesterol with statins and/or diet [22,60,61]. In this case, the fact that the biomarker can be modified by diet or medication does not detract from its value as a risk factor for these late stage cardiovascular diseases. In contrast, using CRP as a risk factor for heart disease is confounded when the individual has a systemic inflammatory disease and it is generally recommended that this biomarker not be used in this subset of the population [22].

As we consider the spectrum of potential endophenotypes for AMD including those that are potentially modifiable biomarkers, it is clear that testing these hypotheses is not the same as evaluating these indicators as AMD-related phenotypes. Looking for differences with these “markers” between AMD cases and controls may help to establish if they are associated with AMD (the first requirement for an endophenotype) but doesn’t evaluate the requirement than an endophenotype is detectable in individuals with an increased risk for developing AMD who have not yet demonstrated clinical disease.

We recently shifted our longitudinal study of at-risk AMD family members and their spouses to specifically evaluate potential structural and functional alterations as potential endophenotypes for AMD. As suggested above, we incorporated the use of the genetic profile to determine a risk score in unaffected family members. We then tested the association between these risk profiles and dark-adapted rod- and cone-mediated threshold sensitivities and any subtle structural changes (before the appearance of drusen) that may be observed by optical coherence tomography.

## 2. Experimental Section

### 2.1. Subjects

Ninety-eight subjects (mean age = 59.8 ± 4.3 years, range 51 to 69 years) participated in this prospective study. Subjects were recruited based on the presence of a family history of AMD in one or both parents. All subjects underwent a complete ophthalmological exam by a retina specialist (MBG) and were segregated based on funduscopic appearance and OCT measures as normal (not AMD) or abnormal (AMD). We first performed a volume scan consisting of 61 full-width (30°) scan lines, each consisting of the average of 9–15 high resolution (HR) scans. These images were carefully examined to determine whether drusen, the hallmark of AMD, was present in either eye and was used primarily to classify the subjects as having a normal or AMD retina. Short-wavelength and near-infrared autofluorescence images were also examined to assess alterations that might be characteristic of AMD. Because these subjects were recruited based on a family history of AMD, but without visual symptoms and/or a clinical diagnosis of AMD, the majority of the participants had a normal fundus appearance (42 subjects).

Because our focus in this study was on examining structural and functional biomarkers that might be predictive of the development of AMD, those with a diagnosis of AMD of any type were not studied further. Group characteristics are shown in Table 1. The study was carried out with approval of the UCLA and University of Pittsburgh Institutional Review Boards (IRB), informed consent was obtained from all subjects prior to participation, and the study was conducted in accordance with regulations of the Health Insurance Portability and Accountability Act of 1996 (HIPAA).

**Table 1:** List of single nucleotide polymorphisms (SNPs) used to calculate the age-related macular degeneration (AMD) genetic risk score and the genotyping success rate.

#	Marker	Genotyping Success Rate	Chromosome	Location	Gene
1	rs10737680	0.979	1	196,710,325	*CFH*
2	rs6795735	0.985	3	64,719,689	*ADAMTS9-AS2*
3	rs13081855	0.9625	3	99,762,695	*COL8A1*
4	rs4698775	0.9985	4	109,669,323	*CFI*
5	rs3130783	0.958	6	30,806,580	*C2-CFB-SKIV2L*
6	rs429608	0.997	6	31,962,685	*SKIV2L*
7	rs943080	0.997	6	43,858,890	*VEGFA*
8	rs3812111	1	6	116,122,572	*COL10A1*
9	rs13278062	1	8	23,225,458	*TNFRSF10B-LOC389641*
10	rs334353	0.9925	9	99,146,083	*TGFBR1*
11	rs10490924	0.9895	10	122,454,932	*ARMS2*
12	rs9542236	1	13	31,245,188	*B3GALTL*
13	rs8017304	1	14	68,318,360	*RAD51B*
14	rs920915	0.997	15	58,396,268	*LIPC*
15	rs1864163	0.994	16	56,963,321	*CETP*
16	rs4420638	0.964	19	44,919,689	*APOC1/APOE*
17	rs8135665	0.994	22	38,080,269	*SLC16A8*

### 2.2. Genetic Analysis

Our cohort of “at-risk” participants was screened for variants in genes commonly associated with AMD, including *ARMS2* and *HTRA1*, two genes in strong linkage disequilibrium on chromosome 10q26, as well as genes of the complement system. Genotyping of 743 individuals which included the subset of individuals in this phenotype-genotype study was done with an iPLEX panel of 88 SNPs that have been selected for known genes associated AMD and with the complement activation cascade, including *CFH* and *CFH*-related genes, *CFI*, *C2/BF*, and *C3*. The raw data was run through dbVor, a database system developed by Baron and Weeks for importing, editing and exporting genotype data, to generate the data in Mega2 format [62]. dbVor’s statistics excluded 3 markers with no measurable genotypes. Mega2 was used to convert the data to PLINK [63]. SNP’s with genotyping success rates <0.9 were removed (18 SNPs) and individuals with genotyping success rates of <0.9 were also excluded. The genetic loci for the 17 of the 19 risk SNPs that were included in the computation of the genetic risk score are described elsewhere [64]. The two markers, rs2230199 and rs5749482, were removed because of excessive genotyping failure rates, 28.94% and 10.36%, respectively. All of the markers used in the AMD genetic risk score calculation showed no evidence of violation of the Hardy Weinberg Equilibrium.

(1) $${\hat{\beta }}_{j}=\beta_{j}\text{/}\sum_{k=1}^{17}\beta_{k}$$

(2) $$S_{i}=\sum_{j}{\hat{\beta }}_{j}g_{ij}$$

The 17 loci can distinguish cases and controls relatively well (area under the receiver operator curve (AUC) = 0.74) [64]. The risk score computation used the formula from the Nature Genetics paper (see below) [63]. In their approach, they normalized the betas by dividing them by the sum of the betas. The score is then the sum over all the observed genotypes of the normalized beta multiplied by the number of risk alleles. This score ranges from zero to two.

Individuals, whose DNA yielded low quality genotyping scores or more than two missing genotypes, were excluded from these analyses. The cohort of participants that were classified as normal based on a normal OCT and fundus exam, were further sub-divided into two groups based on the presence or absence of AMD risk alleles.

### 2.3. Microperimetry

Fundus-guided sensitivity measures were obtained with a Nidek MP-1S microperimetry system (Nidek Technologies, Italy). Sensitivity measures were obtained at discreet locations (0°, 2°, 5°, 10°, 14°) spanning the horizontal and vertical meridian of both eyes. Stimulus size was equivalent to Goldmann V (2.0°) with flash duration of 200 msec. A 4-2 thresholding strategy was employed in derivation of sensitivity measures. Participants were dilated (0.5% tropicamide hydrochloride and 1% phenylephrine hydrochloride) and dark-adapted for at least 30 minutes in a light-tight room prior to data collection. An array of four small crosses arranged in a diamond was used to align and maintain fixation, each cross 3° from the anatomical fovea. After instruction and training, sensitivity was first measured using a long-wavelength “red” stimulus (Edmund Optics, red dichroic filter, NT30-634). The red series was presented first to establish locus of fixation and reliability estimates. A 1.0 neutral density filter could be inserted into the optical pathway to extend the range of flash intensities to approximately 30 db. Immediately after, sensitivity to a short-wavelength “blue” stimulus (Edmund Optics, blue dichroic filter, NT30-635) was measured in exactly the same locations as those for the red flashes. Up to a 3.0 log unit neutral density filter could be inserted into the optical pathway to extend the range of flash intensities to approximately 50 db. In instances where the threshold spanned the boundary between two ND filters, the field was first measured with the denser filter and then replicated with the next less dense filter. Only in case where the threshold was indeterminate at one filter density was the lower density filter used. All starting eyes were counter-balanced across all participants, except in cases of maculopathy where the better eye was tested first.

### 2.4. Optical Coherence Tomography

Retinal structure for all participants was measured with spectral-domain optical coherence tomography (sdOCT) (Heidelberg Engineering, Germany). We first performed a volume scan consisting of 61 full-width (30°) scan lines, each consisting of the average of 9–15 high resolution (HR) scans. These images were carefully examined to determine whether drusen was present in either eye. We then recorded a high-resolution vertical and horizontal scan centered on the anatomical fovea. These images were imported into custom software (Igor Pro; WaveMetrics, Inc., Portland, OR, USA) to segment the different retinal layers as previously described [65,66,67,68]. For the purposes of this study we report thickness measures for Outer Limiting Membrane (OLM)—Bruch’s membrane, Outer Nuclear Layer (ONL), and full retinal thickness.

In addition to the thickness measures, we also derived light reflectance profiles (LRPs) across the retina to measure the relative intensities of the bands seen on the OCT. A high resolution, 30° vertical scan centered on the fovea was used for analysis. Images were imported from native HEYEX software into NIH’s ImageJ (http://imagej.nih.gov/ij, v1.46r, 64-bit) and cropped to include only the OCT scan. After the image was calibrated to match the original scan’s spatial resolution, a 1 mm wide section of the retina was sampled from Bruch’s membrane (BM) to the inner limiting membrane (ILM). Sections centered 1 mm superior and inferior of the fovea were taken. Reflectance profiles were created for each section; values were then divided by the mean gray-scale intensity of the section, thus normalizing plots across patients. To account for varying retinal thicknesses, total retinal thickness was normalized to 1.0 for each eye.

## 3. Results

### 3.1. Characteristics of the Study Population

Ninety-eight subjects were recruited for this study. All participants underwent a complete ophthalmological exam by a retina specialist (MBG) and were segregated based on funduscopic appearance and OCT measures as normal (not AMD) or abnormal (early or intermediate AMD). Fundus grading and AMD classification was done independently by two graders using the Beckman AMD Classification System. Eight of the 98 subjects were found to have significant epiretinal membranes (ERM) in one or both eyes. These eyes were excluded from further study. Of the 98 subjects, 63 were found to have no clinical signs of AMD, 18 subjects were found to have early AMD (medium-sized drusen (diameter >63μ and ≤125μ) but without pigmentary abnormalities) and 13 subjects were found to have intermediate AMD (large drusen (diameter >125μ) or with pigmentary abnormalities associated with at least medium-sized drusen). No participants with late AMD (lesions associated with neovascular AMD or geographic atrophy) were identified in this study. Two subjects with funduscopic changes that were not consistent with AMD were excluded.

### 3.2. Genetic Risk Scores are Associated with Fundus Appearance

Genetic risk profiles for AMD based on 17 key SNPs as identified by the International AMD genetic consortium were determined for each participant. Individuals, whose DNA yielded low quality genotyping scores or more than 2 missing genotypes, were excluded from these analyses. Genetic risk scores were obtained for 69 participants and ranged from 0.61 to 1.62. As shown in Table 2, there was a clear association between AMD risk score and AMD categories based on retinal structural changes (no *t*-test comparing the risk scores among the three AMD categories had *p*-values >0.018).

**Table 2:** Fundus grading and AMD classification using the Beckman AMD Classification System for the 69 individuals with AMD genetic risk scores. Genetic risk profiles for AMD were based on 17 key SNPs as identified by the International AMD genetic consortium.

Fundus Grading based on OCT Imaging	*N*	Mean Risk Score (±1 SD)
Normal (no drusen)	43	1.07 (0.20)
Early AMD (few small drusen)	12	1.24 (0.12)
Intermediate AMD (multiple large drusen)	14	1.41 (0.19)

### 3.3. The AMD Risk Score and Age Correlate with Clinical Findings

**Figure 1:**
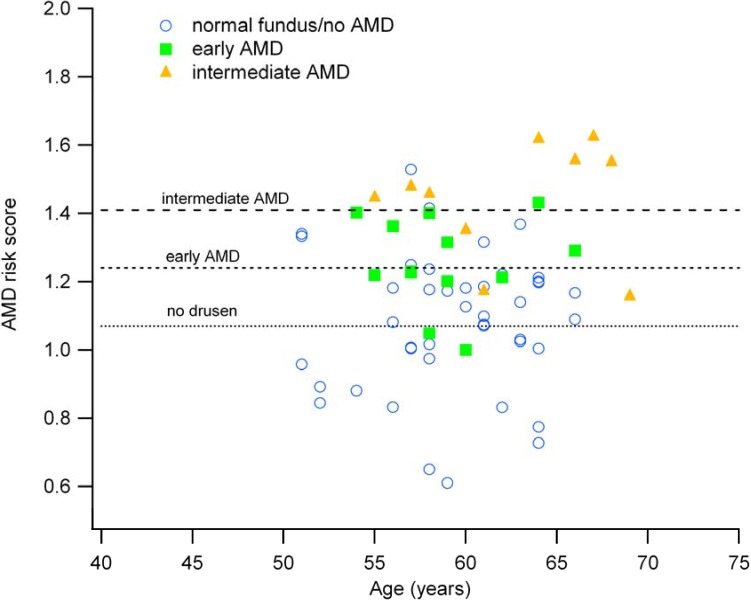
The association between age and AMD genetic risk score (*n* = 69). Each data point represents a separate individual and is color-coded for AMD classification. The horizontal dashed lines indicate the mean risk scores for normal (no drusen), early AMD and intermediate AMD.

Figure 1 displays the associations of age and AMD risk scores with respect to OCT-based evidence of early AMD (any small drusen), intermediate AMD (one or more large drusen), or no fundus lesions. Although considerable variability is apparent, individuals with intermediate AMD are both older and tend to have higher risk scores than those with early AMD or no lesions. Only a single person (age 57) with an AMD risk score greater than 1.4 had a completely normal fundus. Though more data are essential, the age distribution between 50 and 66 appears to be an appropriate range for endophenotype analyses and for defining progressive retinal anatomic changes.

### 3.4. Individuals with High-Risk Genetic Profiles Do Not Necessarily Exhibit Reductions in Scotopic and Photopic Sensitivity

**Figure 2:**
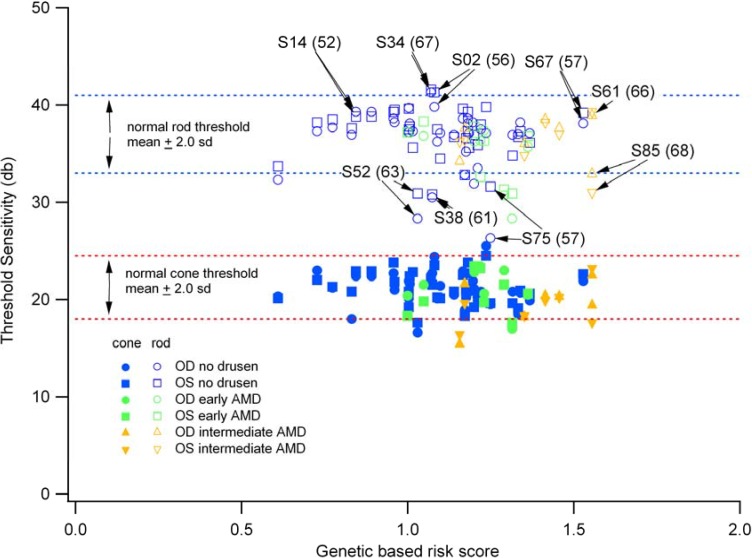
Final thresholds measured psychophysically with dark-adapted microperimetry. Right (OD) and left (OS) eyes are shown separately as circles and squares, respectively. Normal ranges (±2.0 SD) are shown as dashed lines for rod-mediated function (blue lines) and for cone-mediated function (red lines).

The majority of individuals with genetic risk but with normal fundus appearance and no drusen demonstrated normal scotopic and photopic threshold sensitivity (see Figure 2). However, we identified three individuals, subjects S38, S52, and S75, whose scotopic sensitivities were clearly reduced from normal but with cone threshold sensitivities that were within normal limits, although toward the lower limits of normal. This finding is consistent with numerous studies of dark-adapted visual function in early AMD showing greater rod- than cone-dysfunction (e.g., [54]). As a measure of the relative loss of rod function, a ratio of rod to cone sensitivity was formed. For the cohort with normal rod and cone sensitivity, the ratio was 1.77 (±0.1), whereas for the three outliers, the average ratio was 1.5 (±0.18). The average genetic risk score was comparable between the two groups at 1.07 (±0.24) and 1.12 (±0.12), for the normal and reduced sensitivity cohorts, respectively. However there is considerable variability in both rod- and cone-mediated thresholds among the participants that might be relevant to the phenotypic characterization of subtypes of AMD.

### 3.5. Reduced Rod-Mediated Threshold Sensitivity in Patients with Normal Fundus Is Not Clearly Accompanied by Retinal Structural Changes in the Absence of Drusen

**Figure 3:**
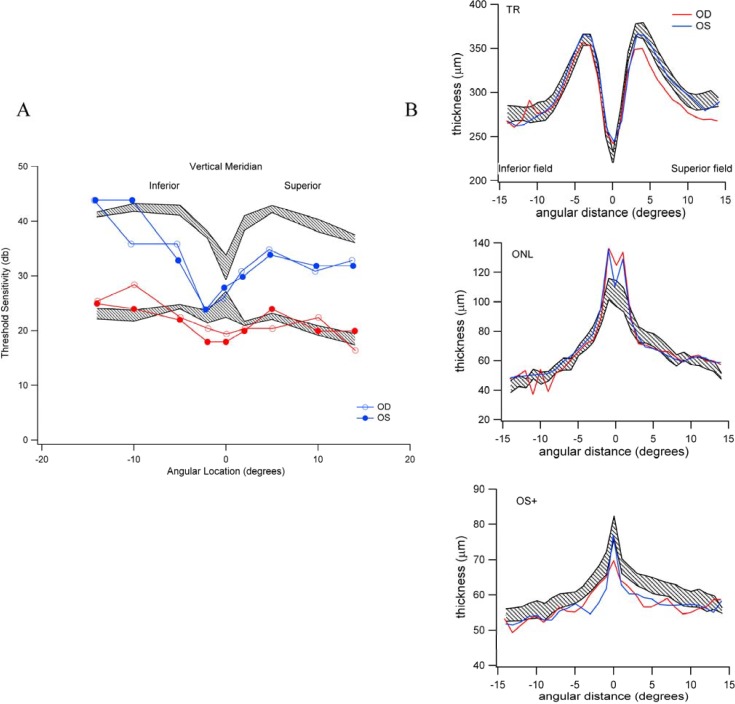
Structural and functional measurements for subject S38 (see Figure 2) with normal appearing retina and no drusen. A: Rod- (blue) and cone- (red) mediated threshold sensitivities at discreet locations along the vertical axis bisecting the anatomical fovea. Normal sensitivity limits are defined by the shaded areas for rods (upper shaded area) and for cones (lower shaded region); B: Retinal thickness (TR) measurements along the same vertical axis for the TR (upper panel), outer nuclear layer (ONL) (middle panel) and the OS+ (lower panel). In each panel, the normal limits are defined by the shaded regions.

We then asked whether the loss of sensitivity observed psychophysically was accompanied by retinal structural change as measured by OCT. For this purpose, we studied the three patients with significantly reduced rod sensitivity (S38, S52 and S75) and compared retinal layer thickness measures with three patients with normal to above average sensitivity (S14, S34 and S02) (see Figure 2). The average age of patients in the two groups was 60.3 and 58.3 years, respectively. First, we segmented vertical sections of the high-resolution OCT images to obtain thickness measures for the OS+ (defined as the thickness from the ISe to Bruch’s membrane), the outer nuclear layer (ONL), and total retinal thickness (TR). The vertical section bisected the anatomical fovea. Average thickness measures for the normal sensitivity group were 55.3 (+4.0), 63.8 (+2.43), and 287.7 (+7.6) μm for OS+, ONL, and TR, respectively. The corresponding measures for the three patients with low sensitivity were 58.2 (+2.5), 68.6 (+4.8), and 298.8 (+10.7) μm. An example of the imaging segmentation and scotopic/photopic threshold regional measurements are shown in Figure 3. Surprisingly, all three measures demonstrate a modest thickening of the measured layers in the low sensitivity group compared to the normal sensitivity, although there is considerable overlap in the distributions of measures. Whether these modest changes in retinal layer thicknesses represent early retinal changes that predict the development of early AMD is not known. However, abnormal thickening and thinning of the photoreceptor layer has been previously reported in intermediate AMD [69].

### 3.6. The Relative Brightness of the Bands Seen on OCT in Those Individuals with Reduced Scotopic Sensitivity Is Not Reduced?

**Figure 4:**
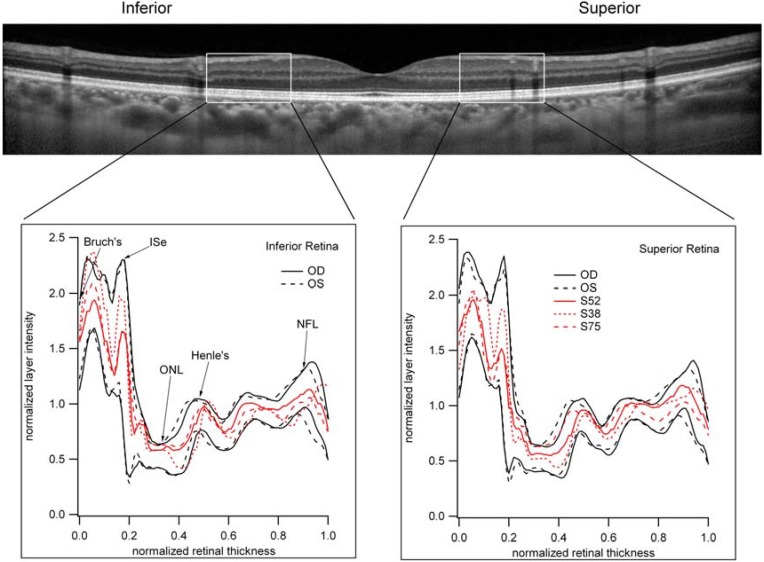
Light reflectance profiles (LRP) across retinal layers as imaged by OCT. A 1 mm wide section of the retina (shown as the rectangle in the OCT image below) was sampled from Bruch’s membrane (BM) to the inner limiting membrane (ILM). Sections were centered 1mm superior and inferior of the fovea. In the bottom panels, the normal range of LRP are defined by the solid black lines. The LRPs for the three patients (S38, S52 and S75) with significantly reduced retinal sensitivity and normal appearing retina are shown as red lines. Note that the LRP for these representative subjects are well within normal limits.

A prominent feature in retinal disease is the disruption of a reflectant band now referred to ellipsoid band (ISe) of the inner segment [70]. This highly visible band has been shown to be absent or disrupted in macular holes [71,72] acute zonal occult outer retinopathy (AZOOR) [73], epiretinal membranes [74], age-related macular degeneration [75,76,77,78], and retinitis pigmentosa [66]. Moreover, a recent study has suggested that both rods and cones contribute to the integrity of this band, becoming less intense in disease affecting primarily cones, and entirely absent when rod- and cone photoreceptors are absent [66]. These findings may provide a novel marker of disease severity in pre-symptomatic AMD. To investigate this possibility, we measured the light reflectance profiles (LRPs) in the midperipheral retina (see Figure 4) to measure the relative intensities of the bands seen on the OCT in those patients (S38, S52 and S75) with abnormal scotopic sensitivity, but with otherwise normal-appearing retina. As can be seen in Figure 4, there were no significant differences in the LRP in these patients compared to those with normal sensitivity (show as black lines are the upper and lower limits for normal).

## 4. Conclusions

In this minireview and research communication, we have discussed the concepts of phenotypes, endophenotypes and biomarkers in the context of age-related macular degeneration. We have attempted to show that the genetic risk profiles based on common SNPs can serve as a valid endophenotype for AMD, particularly in younger, individuals with minimal or no clinical findings of AMD. The high correlations of early and intermediate AMD (as detected by spectral domain OCT) with the AMD genetic risk profile lends strong support to this assertion. These genetic risk profiles allow us to test hypotheses that specific biochemical changes, imaging parameters and/or psychophysical measurements can be tested for abnormalities that precede the onset of clinical findings of AMD. Others [79] have also begun to employ this strategy. While a number of abnormalities of retinal structure and function have been implicated as AMD phenotypes, we have shown that some of these parameters, such as scotopic thresholds, do not fulfill the criteria as endophenotypes for AMD and do not contribute to our ability to refine the risk models that we currently have available. However this is not intended to imply that other parameters such as dark adaptation kinetics and rod- and cone-mediated temporal acuity may not prove to be validated in the future. There is considerable value to be gained from continuing studies of these phenotypes to better define subtypes of AMD and the prognosis of those who have clinical evidence of AMD.

The genetic risk model for AMD may be slightly enhanced by future discoveries of both common and rare variants for AMD, but it is unlikely that it will achieve sufficient sensitivity and specificity to be used in isolation in a presymptomatic, at-risk pool of individuals. Family history also can play a useful role in assessing a patient’s risk of AMD but it is not sufficient by itself. In our cohort, that was drawn from individuals with a positive family history for AMD and also genotyped for a number of AMD-risk related SNPs, we observed only a single participant with an AMD risk score greater than 1.4 who had a completely normal fundus. However this discrimination ability will be less clear as more and younger at-risk individuals undergo high-resolution retinal imaging.

Commercially available tests such as ArcticDX™ and Retnagene™ have already incorporated clinical findings, age and smoking exposure to dramatically improve the predictive ability of their models. However these tests are not appropriate for individuals who have no evidence of AMD and who might wish to know if they should take steps to lower their risk of developing AMD. Genetic testing for these individuals is not appropriate since none of the risk reduction approaches for AMD (nutrient supplements, avoidance of smoking, healthy diets and exercise) are associated with sufficient additional costs or risks to employ genetics-based coercive and/or selective strategies.

The potential value of serum and/or retinal biomarkers to monitor pre-disease risk and response to therapy is undisputed but remains elusive. It will take considerable effort to validate these markers, particularly those that are subject to fluctuations in association with other acute and chronic conditions. Prospective, long-term studies that are focused on high-risk cohorts and lasting 5 to 10 years (similar to the ARED Study) will be required to test and confirm the utility of these markers. Such studies are unlikely to be funded by NIH at this time but it may be possible to compile sufficient preliminary data with smaller, short-term retrospective studies to warrant a clinical trial. Any current or planned clinical trials for AMD therapy which are evaluating disease progression should seriously consider acquiring serum and/or plasma samples as well as DNA for these investigations so that one can use the careful phenotypic analyses and documentation of incident disease and progression for association testing.
